# Biomass-Derived Carbon Heterostructures Enable Environmentally Adaptive Wideband Electromagnetic Wave Absorbers

**DOI:** 10.1007/s40820-021-00750-z

**Published:** 2021-12-04

**Authors:** Zhichao Lou, Qiuyi Wang, Ufuoma I. Kara, Rajdeep S. Mamtani, Xiaodi Zhou, Huiyang Bian, Zhihong Yang, Yanjun Li, Hualiang Lv, Solomon Adera, Xiaoguang Wang

**Affiliations:** 1 Jiangsu Co-Innovation Center of Efficient Processing and Utilization of Forest Resources, Nanjing Forestry University, Nanjing, 210037 People’s Republic of China; 2 Willian G. Lowrie Department of Chemical and Biomolecular Engineering, The Ohio State University, Columbus, OH 43210 USA; 3 Institute of Materials Research and Engineering, Agency for Sciences, Technology and Research, Singapore, Singapore; 4 Sustainability Institute, The Ohio State University, Columbus, OH 43210 USA; 5 Department of Mechanical Engineering, University of Michigan, Ann Arbor, MI 48109 USA

**Keywords:** Electromagnetic dissipation, Carbon heterostructure, Environment adaptability, Bamboo, Lignocellulose

## Abstract

Although advances in wireless technologies such as miniature and wearable electronics have improved the quality of our lives, the ubiquitous use of electronics comes at the expense of increased exposure to electromagnetic (EM) radiation. Up to date, extensive efforts have been made to develop high-performance EM absorbers based on synthetic materials. However, the design of an EM absorber with both exceptional EM dissipation ability and good environmental adaptability remains a substantial challenge. Here, we report the design of a class of carbon heterostructures via hierarchical assembly of graphitized lignocellulose derived from bamboo. Specifically, the assemblies of nanofibers and nanosheets behave as a nanometer-sized antenna, which results in an enhancement of the conductive loss. In addition, we show that the composition of cellulose and lignin in the precursor significantly influences the shape of the assembly and the formation of covalent bonds, which affect the dielectric response-ability and the surface hydrophobicity (the apparent contact angle of water can reach 135°). Finally, we demonstrate that the obtained carbon heterostructure maintains its wideband EM absorption with an effective absorption frequency ranging from 12.5 to 16.7 GHz under conditions that simulate the real-world environment, including exposure to rainwater with slightly acidic/alkaline pH values. Overall, the advances reported in this work provide new design principles for the synthesis of high-performance EM absorbers that can find practical applications in real-world environments.

**Supplementary Information:**

The online version contains supplementary material available at 10.1007/s40820-021-00750-z.

## Highlights

- A novel, non-porous carbon structure was obtained through pyrolysis of biomass heterostructures consisting of cellulose and lignin.
- The novel class of biomass-derived carbon materials exhibit an enhanced electromagnetic (EM) loss capability due to the nano-antenna structure created by in-situ growth of carbon nanofibers on carbon nanosheets.
- The designed carbon materials exhibit good hydrophobicity and acid/base resistance, suggesting a stable EM absorption performance in diverse environmental conditions, thus making it a good candidate for real world conditions.

**Supplementary Information:**

The online version contains supplementary material available at 10.1007/s40820-021-00750-z.

## Graphical abstract

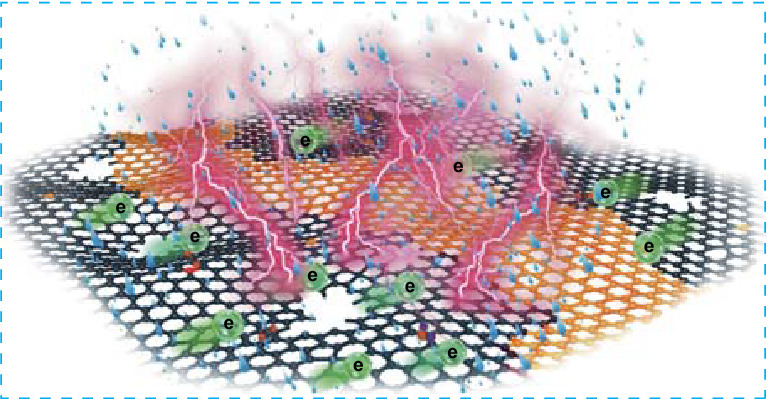

## Introduction

Wireless communication techniques kick-started a grand technical revolution that has significantly improved the quality of human life. However, the frequent utilization of wireless electronics has led to the prevalence of electromagnetic (EM) pollution, which now ranks fourth after water, air, and noise pollution [1–3]. To mitigate EM pollution, EM absorbing materials have attracted lots of attention for their ability to convert ambient EM waves into Joule heat [4–6]. Extensive efforts have been made in the past to develop EM absorbers with wideband EM absorption [7–9]. However, most of the current EM absorbing materials, including magnetic materials [10, 11], metal oxides [12, 13], and sulfides [14, 15], cannot be used in real-world environments due to their poor environmental adaptability, poor water resistance, and temperature sensitivity [16, 17]. For example, EM absorbers are often used as coating layers in outdoor conditions. Thus, EM absorbers resistant to water and acidic conditions are highly desirable to avoid degradation in performance after exposure to acid rain. Although polymeric absorbers have been shown to possess high hydrophobicity, their low dielectric loss leads to poor EM absorption performance [18, 19]. Thus, the development of high-performance EM absorbers with good environmental adaptability will be beneficial for real-world applications.

Recently, carbon-based materials, which can be categorized into materials with a high degree of graphitization (e.g., graphene, metallic carbon nanotubes (CNTs), and graphite) and moderate/low degree of graphitization [20–22], have attracted significant attention for their use as EM absorbers based on four main reasons. First, carbon materials are naturally resistant to acidic mediums due to the strong covalent bonds between the carbon atoms [23–25]. Second, most carbon materials are either non-graphitized or graphitized to only a limited degree [26–28], resulting in the prevalence of hydrophobic bonds, which confer the material with hydrophobic properties. Third, carbon materials' intrinsic conductivity and dipole dependence enhance their EM absorption performance [29–32]. Lastly, carbon materials have ultralow density and are naturally abundant compared to synthetic metallic and polymeric materials [33–35]. Based on the above-stated properties of carbon materials, it is evident that carbon-based materials can serve as promising candidates for making EM absorbers for outdoor applications. However, the realization of this potential remains elusive due to the following reasons. First, previous studies have shown that the ultrahigh dielectric value of highly graphitized carbon materials causes poor impedance matching ability, resulting in poor EM absorption [36–38]. Second, it is difficult to achieve effective, wideband EM absorption in moderate/low-graphitized carbon materials, even with the good impedance matching. Third, carbon materials are produced using a chemical method that inevitably produces a high ratio of hydrophilic bonds (e.g., –OH, –COOH, and –COH), affecting their hydrophobicity and acid resistance. Lastly, although previous studies have shown that the performance of EM absorbers is affected by the chemical component and nanostructure [39, 40], the manipulation of carbon materials' structure to tune the EM absorption has been largely unexplored. The above-stated limitations have prevented the development of weather-resistant carbon material-based EM absorbers.

Carbon materials derived from biomass such as bamboo have been extensively used as electromagnetic wave absorbers due to their low cost, simple synthesis procedure, and potential dielectric loss ability [41, 42]. While promising, current biomass-derived carbon materials cannot exhibit a wideband EM absorption at a thin thickness (< 2.0 mm) due to their limited EM loss capability [43]. As an alternative strategy, carbon material-based composites consisting of biomass-derived carbon materials and other components, such as magnetic metals, transitional metal oxides, etc., have attracted growing attention, aiming to enhance and incorporate other loss abilities [44–46]. Although the carbon material-based composite approach shows some promise, the numerous intrinsic merits of single-component carbon material-based EM absorbers, such as ultralow density and good chemical stability, are lost. Therefore, designing biomass-derived carbon materials-based EM absorbers with lightweight, wideband, and thin thickness remains challenging. The key design challenge is how to enhance the poor dielectric loss ability and the poor hydrophobic property of the carbonized biomass materials, which are caused by (1) the intrinsic porous structures derived from the characteristic morphology of their precursors, (2) the commonly used, low carbonization temperature (i.e., 600–800 °C), and (3) the presence of hydrophilic functional groups at the surface of the carbonized materials.

Herein, we report the synthesis of graphitized biomass-derived carbon materials (GC) with different structures and graphitization degrees through pyrolysis of bamboo-derived lignocellulosic nanofibers (LCNFs). By characterizing the composition and crystallinity of the graphitized carbon materials using X-ray diffraction (XRD) and Raman spectroscopy, we find that the ratio of cellulose to lignin plays a critical role in the nanostructure formed by the graphitized bamboo-based carbon materials. Cellulose/lignin ratios of 5:1 and 8:1 lead to the coexistence of nanofibers with high crystallinity and nanosheets with high-density defects, whereas ratios of 4:1 and 30:1 give rise to only nanosheet structures. Importantly, graphitized carbon materials with the cellulose to lignin ratio of 8:1 exhibits the broadest effective frequency bandwidth ($f_{{\text{E}}}$, 4.2 GHz), the lowest reflection loss value (${\text{RL}}_{\min }$, − 51.0 dB), the thinnest matching thickness (1.95 mm), good hydrophobicity, and acid/alkali corrosion resistance and proved to be the most ideal EM filler for outdoor applications. Finally, we elucidate the EM absorption mechanism of GCs based on impedance matching, polarization, and conductive loss.

## Experimental Section

### Materials

Bamboo residues were obtained from Zhuangchi Home Technology Co. Ltd., (Jiangxi, China). *p*-toluenesulfonic acid (*p*-TsOH) was an analytical reagent purchased from Aladdin Chemical Co. Ltd., Shanghai, China. We prepared all aqueous solutions with deionized water from a Barnstead Nanopure Diamond Laboratory Water System.

### Synthesis of Lignocellulosic Nanofibers

Natural bamboo residues were steam-exploded at 190 °C for 10 min to yield bamboo fibers. Lignocellulosic nanofibrils (LCNFs) were isolated from the obtained bamboo fibers through hydrolysis of 5 g (oven-dry weight) bamboo fibers, and they were reacted with 100 g of prepared p-toluenesulfonic acid (20–80%, w/w) at 50–80 °C for 30–90 min under continuous stirring of 400 rpm*.* Afterward, 50 mL of deionized (DI) water was added to quench the reaction. The residual solids were separated through filtration under a vacuum and washed with DI water. We tuned the cellulose and lignin content in the residual solids by adjusting the acid concentration, reaction temperature, and reaction time. Subsequently, mechanical fibrillation was carried out by feeding 1% (w/w) residual solid suspension into a high-pressure homogenizer (FB-110Q, Litu Mechanical Equipment Engineering Co. Ltd., Shanghai, China) operating at a pressure of 600 bar five times to produce LCNF. The detailed parameters for the preparation of LCNFs are listed in Table S1.

### Synthesis of Multi-Dimensional Carbon Composites

GCs were prepared by temperature-programmed pyrolysis. Precisely, the prepared LCNFs were placed into a tubular furnace under an N_2_ stream. After purging air by filling N_2_ in the furnace for 30 min, the furnace temperature was increased to 1000 °C at a heating rate of 10 °C min^−1^. After refluxing for 1 h at 1000 °C, the samples were further heated to 1500 °C at a heating rate of 5 °C min^−1^, followed by another 2 h of reflux. Finally, the GCs were collected and cooled down to the ambient temperature under N_2_ protection. The nomenclature of our samples is shown in Table S1.

### Characterization

The nanostructure, morphology, and chemical bonds of the synthesized carbon materials were characterized by atomic force microscope (AFM; Asylum Research MFP-3D Bio AFM, Oxford Instruments Company), scanning electron microscope (SEM; Quanta 200, FEI Company), high-resolution transmission electron microscope (HR-TEM; FEI Tecnai G2 F20 S-TWIN), X-ray diffraction (XRD; Bruker D8 Advance powder X-ray diffractometer), X-ray photoelectron spectroscopy (XPS; AXIS UltraDLD, Shimadzu, Al Ka X-ray source, 150 W), Raman spectroscopy (Renishaw in-via Raman micro-spectrometer, 532 nm laser), and Fourier transform infrared spectroscopy (FT-IR, Thermo Scientific Nicolet 6700).

The cellulose and lignin content of the LCNFs were determined based on the procedure developed by the National Renewable Energy Laboratory, as previously reported. The electromagnetic properties of the samples were determined by the coaxial line method using an Agilent PNA N5224A vector network analyzer with a filler loading rate of 15% (w/w) in paraffin. EM absorbing performance of the specimens was evaluated using the reflection loss (RL) values, which are calculated as:

1 $$Z_{{{\text{in}}}} = Z_{0} \left( {\mu_{r} /\varepsilon_{r} } \right)^{1/2} \tanh \left[ {j\left( {2\pi fd/c} \right)\left( {\mu_{r} \varepsilon_{r} } \right)^{1/2} } \right]$$

2 $${\text{RL}} = 20\log_{10} \left| {\left( {Z_{{{\text{in}}}} - Z_{0} } \right)/\left( {Z_{{{\text{in}}}} + Z_{0} } \right)} \right|$$

where *ε*_*r*_ (*ε*_*r*_ = $\varepsilon^{\prime} - j\varepsilon^{\prime\prime}$, where *j* denotes imaginary part symbol, and $\varepsilon^{\prime}$ $\varepsilon^{\prime\prime}$ are the real and imaginary parts of permittivity) is the relative complex permittivity, *μ*_*r*_ (*μ*_*r*_ = $\mu^{\prime} - j\mu^{\prime\prime}$, where $\mu^{\prime}$ and $\mu^{\prime\prime}$ are the real and imaginary parts of permeability) is the relative complex permeability, *f* is the microwave frequency, *d* is the specimen thickness, *c* is the light velocity, and *Z*_0_ and *Z*_in_ are the characteristic impedance of free space and the input impedance of the absorber, respectively.

Hydrochloric acid or sodium hydroxide was used to prepare weak acid and weak base solutions with pH of 5.6 or 8.5, respectively, to investigate the stability of the EM absorbers under acid/base conditions. The samples were immersed in the respective solutions for 7 days. Afterward, the samples were centrifuged, washed, and dried. The coaxial line method was used to measure the electromagnetic properties, and the corresponding EM absorption parameters were calculated.

## Results and Discussion

### Characterization of Lignocellulosic Nanofibers

**Fig. 1:**
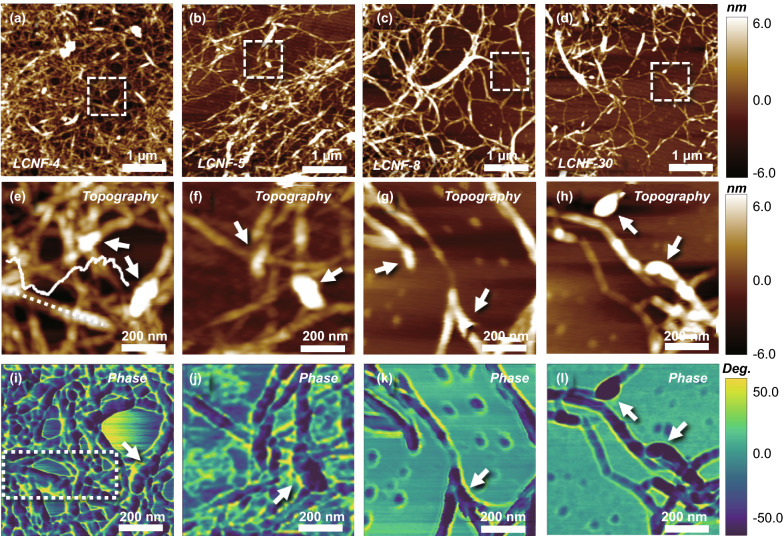
AFM imaging of LCNF morphologies. **a**–**d** AFM topography images of LCNF-4, LCNF-5, LCNF-8, and LCNF-30. **e**–**h** High-resolution AFM topography images of the white dashed boxes in **a**–**d**. **i**–**l** Corresponding AFM phase images of **e**–**h**. The white arrows indicate the adhesive lignin on the surface of the cellulose

As described in the Introduction, the EM absorption performance is determined by the constituent component and nanostructure of the EM absorbers. Previous studies have shown that the direct utilization of the biomass for fabricating EM absorbers results in poor EM absorption due to its poor dielectric loss. The biomass has to be graphitized to enhance its EM performance by improving the conductive loss and polarization behavior. In this study, we sought to develop EM absorbers using a carbonized biomass precursor (lignocellulosic nanofibers (LCNFs)) and tune the EM absorption performance by adjusting the composition and structure of two components, lignin, and cellulose. Specifically, we synthesized several LCNFs with ratios of cellulose to lignin varying from 4:1, 5:1, 8:1, to 30:1. The precursors corresponding to the above ratios were termed LCNFs-4, LCNFs-5, LCNFs-8, and LCNFs-30, respectively. Considering the fact that the direct visualization of soft organic molecules like cellulose/lignin using SEM/TEM is extremely challenging, owing to the possible decomposition of the organic molecules under a high-energy electron beam [47], we characterized the structures of the LCNFs using AFM. The nanofiber structures were observed in all LCNFs, as shown in Fig. 1a–d. As the cellulose composition in the LCNF (the ratio of cellulose to lignin) is increased, the density of nanofibers decreased, and no additional nanostructures were identified. We sought to use high-resolution AFM to explore the specific characteristics of the LCNFs components. In AFM imaging, different components would exhibit distinct appearances upon exposure to the high-resolution alternating current (AC) in the air topography model. As shown in the representative high-resolution AFM images, the backbones of the nanofibers were dark. At the same time, their surfaces were observed to be modified with individual bright clusters (represented by white arrows in Fig. 1e–h). Additionally, the height associated with the bright clusters was larger than that of the backbones, as shown in the inset of Fig. 1e. Moreover, two distinct contrasts were observed in the corresponding phase conversion images (Fig. 1i–l). The above observation reveals that the two biomass components, i.e., cellulose and lignin, form heterojunctions.

Inspection of the above AFM images suggests that one component of the LCNFs possesses a nanofiber structure, whereas the other component possesses an irregular structure. To distinguish the nanofiber heterojunctions, we sought to analyze the molecular arrangement to determine the structure that yields nanofibers and the attached amorphous particles, respectively, because the structure depends on the molecular arrangement and degree of polymerization of the monomers. Intrinsically, lignin comprises three types of monomers, namely p-coumaryl, coniferyl, and sinapyl alcohol [48]. At a low degree of polymerization, the as-prepared lignin are nanoparticles with sizes depending on the degree of polymerization. The nanoparticles would form an interconnected bulk phase as the polymerization increases. The cellulose is fabricated using the linear chains of glucopyranose monomer joined by β-(1,4) glycosidic bonds [49], resulting in a single direction of molecular arrangement. Therefore, the as-prepared cellulose has a 1D nanofiber structure, as shown in the enlarged image marked by a white dashed box in Figs. 1i and S1a–c. The highlighted dots in the topography image (Fig. S1a) correspond to the observed peaks in the height curve (Fig. S1b) and the dark contrast dots in AFM phase image (Fig. S1c). These observations support our hypothesis that lignin molecules are modified on the surface of cellulose fibers with their natural properties maintained after hydrolysis treatment, and that the nanofiber framework in the LCNFs belongs to cellulose and supports lignin growth, which contrasts with the behaviors of pure lignin and cellulose (namely C-lignin and C-cellulose; Figs. S1d–g). These results lead us to hypothesize that the adjacent nanofibers coated by lignin are easier to link with each other through a dehydration reaction between the two –OH groups, resulting in a crosslinked structure at a low cellulose to lignin ratio.

Next, we sought to analyze the crystal structures of these LCNFs. Figure S2 shows the crystal structure and chemical bonds of these LCNFs. We observed two diffraction peaks at ~ 16° and 23°, corresponding to the (101) and (002) crystal planes of cellulose, respectively, as shown in Fig. S2a. C and O were observed in all LCNFs as shown in the XPS spectra in Fig. S2b. We note here that H is a light element and cannot be detected by XPS. We observe that the proportion of O in the samples increases with an increase in the cellulose content. By fitting the high-resolution O_*1s*_ peaks, the spectra for all the LCNFs can be deconvoluted into three peaks at 532.0, 532.9, and 533.9 eV, corresponding to C=O of the lignin, C–O of the cellulose, and phenolic oxygen of the lignin, respectively (Fig. S2c). Next, we used the fitting peak surface area to quantify the cellulose to lignin ratio. We find that as the cellulose to lignin ratio increases from 4:1 to 30:1, the percentage of C–O increases from 76 to 93%, and the total percentage of C=O and phenolic oxygen decreases from 24 to 7%, suggesting the removal of lignin from the LCNFs. As shown in Fig. S2c, we observed three deconvoluted peaks for C_*1s*_ at 283.8, 285.0, and 286.5 eV, corresponding to C=O in lignin, C–C and C–H in holocellulose, and C–O in phenols and ethers of lignin and holocellulose, respectively. As the ratio of cellulose to lignin increases from 4:1 to 30:1, the percentage of C = O decreases from 15 to 7%, which is in agreement with the result of O_*1s*_. The XPS results, combined with the AFM imaging, lead us to draw the following three conclusions. First, LCNFs form a nanofiber heterojunction in which the 1D nanofiber of cellulose serves as the framework for the subsequent lignin coating. Second, the reduced lignin content facilitates the dispersion ability of the nanofibers. Third, the dipolar covalent bonds ratio in the LCNF precursors affects the degree of graphitization after the carbonization.

### Structural Characterization of Biomass-Derived Carbon Materials

**Fig. 2:**
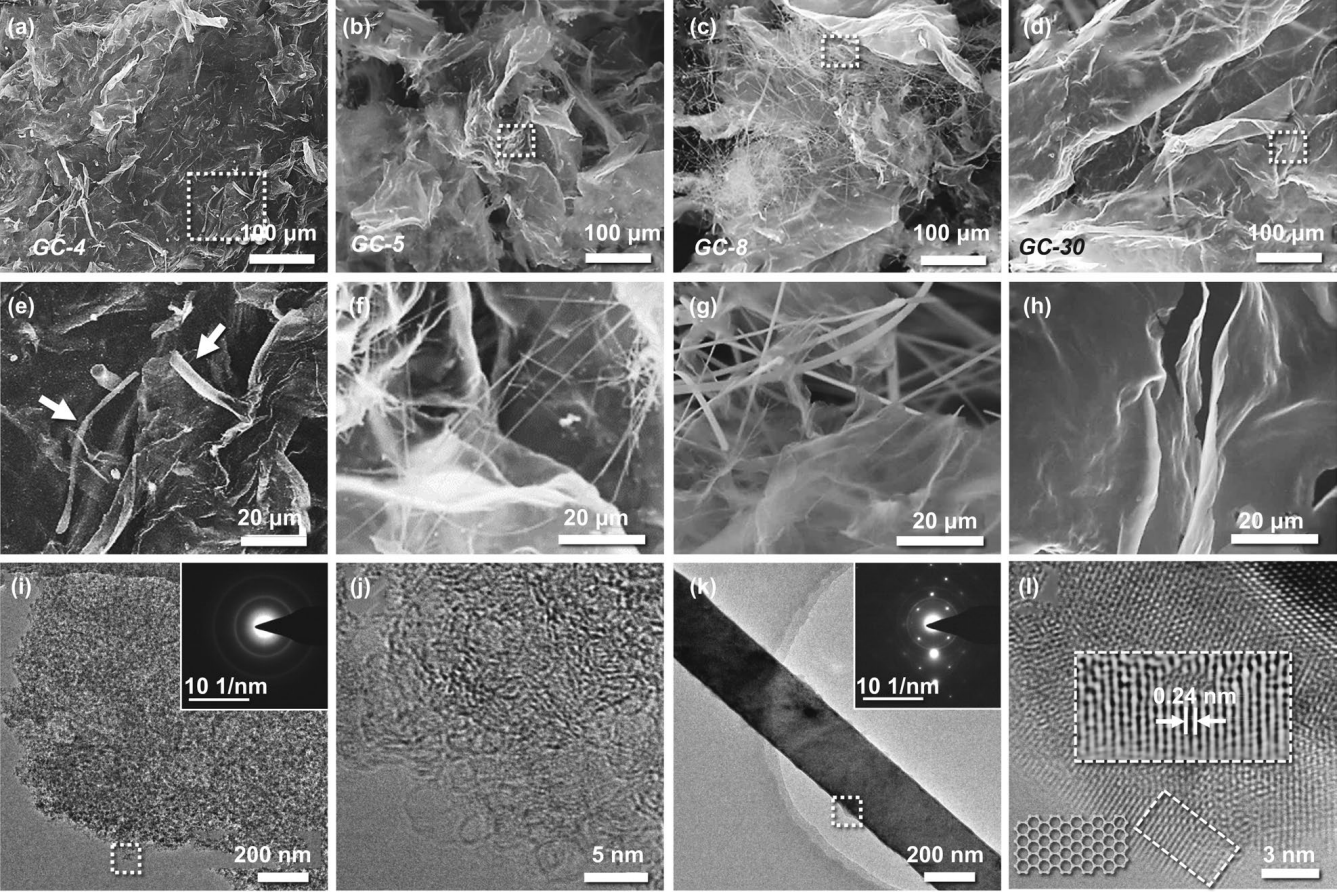
Morphology and crystallinity of GCs. **a**–**d** FE-SEM images of GC-4, GC-5, GC-8, and GC-30. **e**–**h** Enlarged images of the regions marked by white dashed boxes in a-d. The white arrows show the nanofibers embedded in the nanosheets. **i**, **k** Representative TEM images of nanosheets and nanofibers from GC-8. Inset shows the corresponding SEAD pattern. **j**, **l** High-resolution TEM images of the selected areas in **i** and **k**. Inset shows the structure diagram

Past research has reported the conversion of cellulose and lignin from LCNFs into graphitized carbons during carbonization [50]. In our next set of experiments, we carbonized the above LCNFs, i.e., LCNFs-4, LCNFs-5, LCNFs-8, and LCNFs-30, to obtain carbonized materials, namely GC-4, GC-5, GC-8, and GC-30, respectively. The structure of the GC-4, GC-5, GC-8, and GC-30 was examined using FE-SEM, as shown in Fig. 2a–d. The GC-4 and GC-5 samples exhibit thick nanosheet structures with high surface roughness (Fig. 2a, b, e, f). Both the nanofibers and nanosheets were observed in GC-8 (Fig. 2c, g). In addition, GC-30 exhibits only nanosheet structures with a thinner thickness (few nanometers, similar to graphene) compared to that of GC-4 and GC-5 (Fig. 2d, h). To provide more evidence regarding the structural evolution, particularly for nanofibers, we imaged the nanosheets and nanofibers in GC-8 using TEM, high-resolution TEM, and electron diffraction, as shown in Fig. 2i–l. The lattice spacing of the nanosheet structure reveals low crystallinity, which is consistent with the selected electron diffraction pattern (SEAD, inset in Fig. 2i). In contrast, the distinct lattice spacing identified at the edge of the nanofiber was measured to be 0.24 nm, corresponding to the (002) crystal plane of graphene. In addition, the hexagonal lattice obtained from SEAD agrees with that of graphene (inset of Fig. 2l). These results suggest that the nanofibers have a higher degree of crystallinity than the nanosheets. For graphitized carbon, high crystallinity indicates a higher graphitization degree leading to higher dielectric values.

**Fig. 3:**
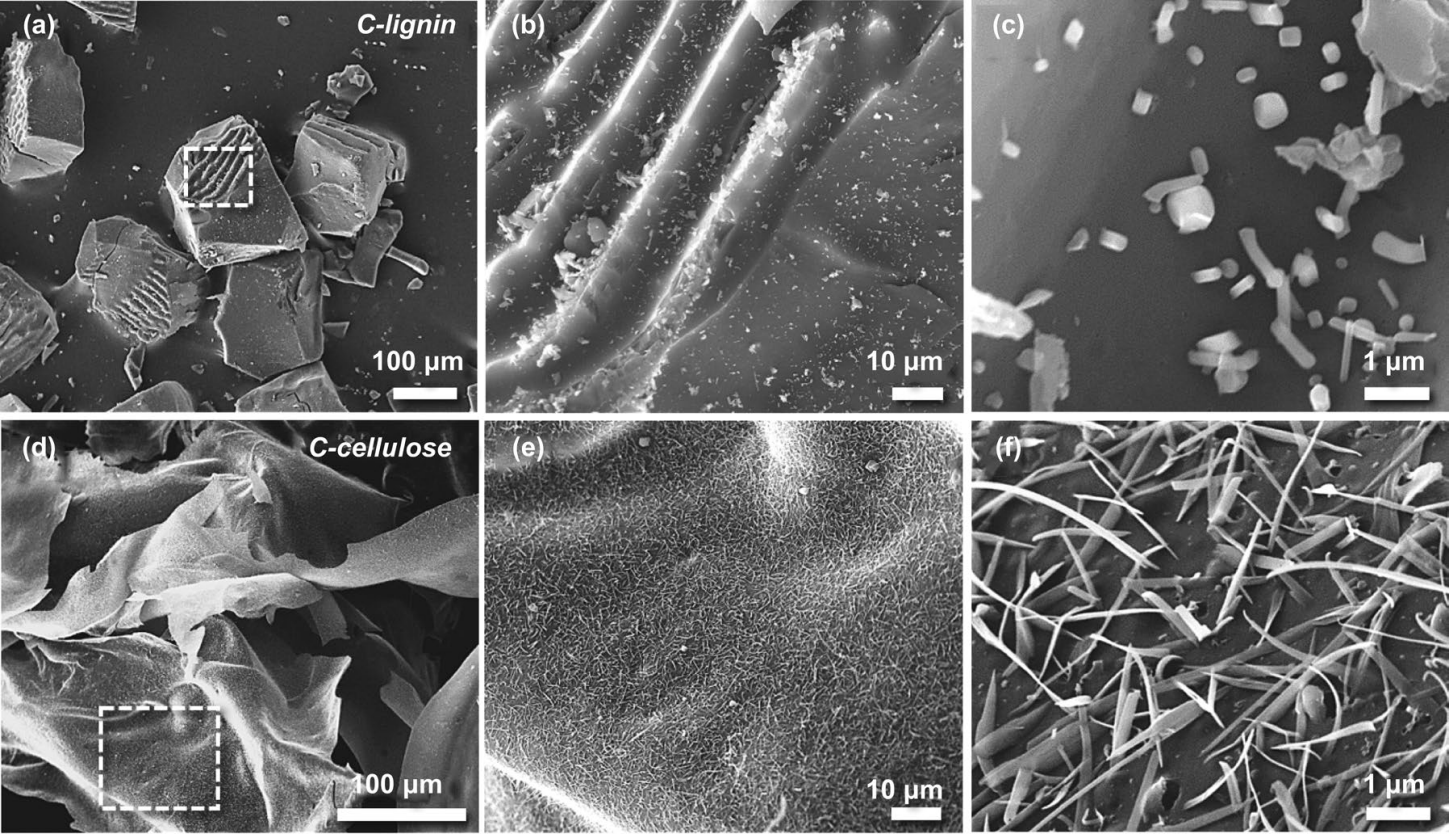
Morphology of carbonized lignin and cellulose. **a**–**c** SEM images of C-lignin. **d**–**f** SEM images of C-cellulose

The above structural analysis raises two questions. First, why are the thickness of nanosheet structures of GC-4 and GC-30 different? Second, why can the nanofiber structure only be observed in GC-5 and GC-8 samples but not in GC-4 and GC-30? Therefore, we performed additional experiments by carbonizing pure lignin and cellulose to gain more insights to answer the above questions. The FE-SEM images in Fig. 3, show the carbonized lignin was transformed into an assembly of nanosheets (Fig. 3a) with a smooth surface (Fig. 3b, c), similar to MXenes [51]. In contrast, the carbonized cellulose was converted into a thin nanosheet structure with a high surface roughness (Fig. 3d–f), similar to the structure of GC-30. Furthermore, we studied the crystal phase structure of the GCs along with carbonized pure lignin and cellulose using XRD. As shown in Fig. S3, the samples exhibit two peaks at 2θ = 25.36° and 43.18°, corresponding to the (002) and (101) planes of graphite-like carbon, respectively (Fig. S3). An additional sharp diffraction peak at 2θ =  ~ 10° was observed in all GC samples, and its intensity decreased with a decrease in the lignin content. According to a previous study [52], this peak indicates the (111) crystal plane of fullerene-like cage structures, attributed to non-hexagonal defects of a corrugated sheet. Thus, the carbonized precursor with a higher ratio of cellulose to lignin suppresses such defects (i.e., non-$sp^{2}$ bonds including $sp^{3}$/$sp$–C–C bonds and dipole O-containing bonds, and the stacking layers), this defect suppression is attributed to graphitization.

Previous research has reported that oxygen-containing functional groups can induce electronic dipole polarization under alternating electromagnetic fields [53]. To provide insights into the structure of GCs, we performed FT-IR measurements to characterize the functional group features of the GCs. As shown in Fig. S4, the absorption band at 3432.6, 1637.2 (sharp band), 1536.9–1295.9 (broadband), and 1066.4 cm^−1^ correspond to the stretching vibration of –OH, C–C, C=O, and C–O groups, respectively. We note here that C=O and C–O groups are from the alkyl aromatic structures. These results suggest that even after carbonization at 1500 °C, the GCs still possess some oxygen-containing functional groups. The XPS spectrum in Fig. S5 also shows that the C–OH and C–OOH groups still exist, although their content decreases with a decrease in the lignin content of the precursors, which are in accordance with the XRD and FT-IR spectra.

**Fig. 4:**
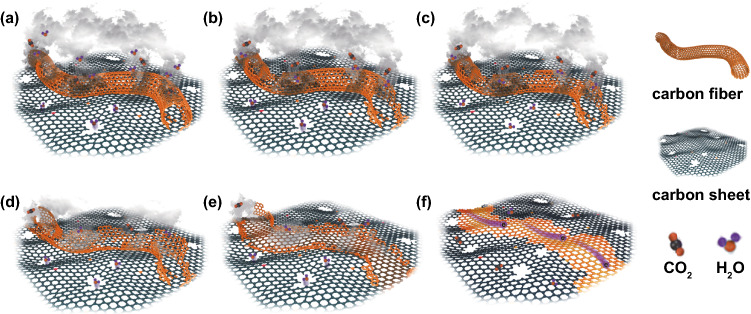
Schematic illustration of the transformation of GCs from heterogeneous structures (e.g., nanofiber and nanosheet) to nanosheet structures

Based on the XRD and FE-SEM images of carbonized lignin and cellulose, we hypothesize that the nanosheet structure in the GC-30 is formed by the self-assembly of carbonized lignin and cellulose. The carbonized cellulose plays a dominant role in the carbon nanostructure. Two possible routes may be responsible for the thick nanosheet structure of GC-4. First, the thick nanosheet originates from the self-assembly of carbonized lignin and cellulose, in which the carbonized lignin plays a dominant role on the carbon nanosheets. However, it is difficult to determine the underlying reason for the high surface roughness. The other possible route is that the carbonized lignin forms the matrix and disperses the carbonized cellulose. The coated lignin on the surface of the cellulose nanofiber can crosslink with adjacent lignin. After carbonization, a thick nanosheet may form with the carbonized nanofiber embedded within the nanosheets. The SEM images show that the crystallinity of the GC-4 nanosheet is lower than that of GC-30, which agrees with the XRD result. We hypothesize that the insertion of the carbonized nanofiber between the nanosheets in the case of GC-5 and GC-8 will be difficult due to the reduced carbonized lignin content, which supports our observation of the presence of nanofibers in GC-5 and GC-8 (Fig. 2f, g). We also comment here that the absence of nanofibers in the thin nanosheets in GC-30 is because of the limited nanofibril cellulose content and the weak dispersion ability caused by the low lignin content (Fig. 4). The large number of hydroxyl groups on the cellulose surface leads to the formation of a densely crosslinked structure via intermolecular hydrogen bonding. During carbonization, the 3D molecular structure of the cellulose would turn to a 2D structure after releasing –OH bonds. Meanwhile, degradation of lignin at high temperatures produces CH_4_ gas and yields carbon atoms, which further grow on the 2D molecular structure of the carbonized cellulose at 1500 °C [54]. Such a combined carbonization and chemical vapor deposition process leads to the formation of intact nanosheets with the decomposed cellulose-derived carbon nanofibers as the main body. Although the defect concentration in GC-5, GC-8, and GC-30 is less than GC-4, layer dislocation or stacking occurs in the assembly process, resulting in a higher ratio of D and G bands in the corresponding Raman spectrum (Fig. S6).

### EM Absorption Behavior of Biomass-Derived Carbon Materials

**Fig. 5:**
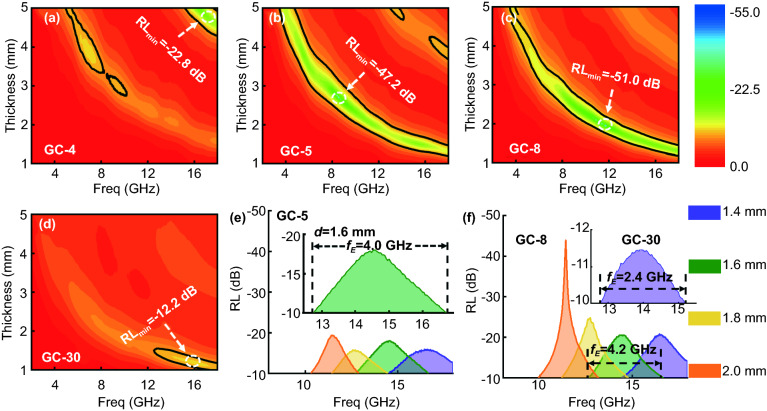
EM dissipation properties of GCs. **a**–**d** 2D colored reflection loss (RL) values of GC-4, GC-5, GC-8, and GC-30. **e** *f*_E_ curves of GC-5 with thickness < 2.0 mm. Inset shows the RL curve with the broadest *f*_E_ of GC-5. **f** *f*_E_ curves of GC-8 and (inset) GC-30 with thickness < 2.0 mm

Next, we sought to investigate the effects of the obtained carbon materials' structure and composition on their EM absorption performance. Figure 5 shows the EM absorption performance of GCs with a broad thickness of 1.0–5.0 mm. EM absorbers are required to exhibit a broad frequency absorption region (termed as an effective absorption region) with a small thickness (commonly less than 2.0 mm) to meet the requirements for commercial applications. As shown in Fig. S7, the minimum RL value $\le - 10 {\text{dB}}$ (termed as effective absorption region, $f_{{\text{E}}}$) with a small thickness (commonly less than 2.0 mm). The minimum RL value (${\text{RL}}_{\min }$) of carbonized pure lignin (C-lignin) is larger than − 10 dB and the ${\text{RL}}_{\min }$ value of carbonized pure cellulose (C-cellulose) is ~ −13.7 dB, as shown in Fig. S7, suggesting their poor EM absorption performances. In contrast, for GCs, GC-4 has a ${\text{RL}}_{\min }$ value of − 22.8 dB at a higher thickness (~ 5.0 mm, Fig. 5a), whereas the $\left| {{\text{RL}}_{\min } } \right|$ of GC-5 increases to 47.2 dB with a corresponding thickness of 2.70 mm (Fig. 5b). The $\left| {{\text{RL}}_{\min } } \right|$ of GC-8 is 51.0 dB with a thickness as thin as 1.95 mm, which meets the requirement for practical application (Fig. 5c), and the $\left| {{\text{RL}}_{\min } } \right|$ of GC-30 reduces to 12.2 dB with a matching thickness of 1.20 mm (Fig. 5d).

As shown in Fig. 5, with an increase in the thickness, the absorption peak shifts to a lower frequency region, which can be described using the following 1/4 wavelength cancelation law:

3 $$t_{{\text{m}}} = nc/4f_{{\text{m}}} \left( {\varepsilon_{r} \mu_{r} } \right)^{1/2} \left( {n = 1, \, 3, \, 5, \ldots } \right)$$

where *t*_m_ and *f*_m_ are the matching thickness and matching frequency for the strongest absorption peak, respectively. Equation (3) indicates that *t*_m_ and *f*_m_ are inversely proportional to each other. We note here that when *t*_m_ and *f*_m_ satisfy this equation, the reflected EM waves both from the air-absorber surface and the absorber-conductive background interface are out of phase by 180°, resulting in extinction at the air-absorber interface and a corresponding minimum RL value. Figure 5e, f plots the curves of *f*_E_ for GCs with thickness below 2.0 mm, which are obtained from their 3D coloring of RL images. The maximal *f*_E_ regions of GC-5, GC-8, and GC-30 are 4.0, 4.2, and 2.4 GHz, with a thickness of about 1.6, 1.6, and 1.4 mm, respectively. We note here that GC-8 possesses the broadest *f*_E_ with a thickness comparable to the recently reported pure carbon-based EM absorbers (listed in Table 1).

**Table 1:** EM absorption performance of different carbon materials

Filler	Matrix	Filler loading	RL_min_ (dB)	*f*_E_ (GHz)	Range (GHz)	References
NRGO/MWCNTs	Paraffin	15%	− 53.3 (2.0 mm)	5.2 (2.0 mm)	11.1–16.3	[55]
NRGO/MWCNTs binary aerogel	Paraffin	10%	− 35.1 (2.0 mm)	3.9 (1.5 mm)	12.1–18.0	[56]
Residual carbon	Paraffin	20%	− 6.8	N/A	N/A	[57]
GCs HCNTs	Paraffin	10%	− 44.8 (3.4 mm)	6.0 (3.1 mm)	8.0–14.0	[58]
B, N-CNTs	Paraffin	10%	− 40.0 (2.0 mm)	4.9 (2.0 mm)	10.5–15.4	[59]
MCHMs	Paraffin	10%	− 51.0 (4.0 mm)	7.1 (2.0 mm)	10.7–17.8	[60]
RGO	Paraffin	3.0%	− 25.6 (4.0 mm)	4.3 (4.0 mm)	8.5–12.8	[61]
CNTs/CF	Paraffin	1.0%	− 44.5 (3.0 mm)	7.4 (3.0 mm)	10.5–17.9	[62]
carbon planar helixes	Paraffin	30%	− 38.0 (3.7 mm)	3.5 (3.7 mm)	11.0–14.5	[63]
GC-8	Paraffin	15%	− 51.0 (2.0 mm)	4.2 (1.6 mm)	12.5–16.7	This work

**Fig. 6:**
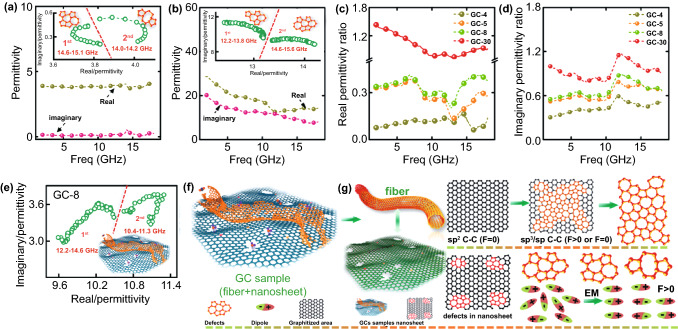
Dielectric properties of GCs. **a**, **b** Permittivity parameters of C-lignin and C-cellulose. Inset shows the corresponding Cole–Cole plots. **c**, **d** The real permittivity ratio and imaginary permittivity ratio of GCs to C-cellulose. **e** Cole–Cole plots of GC-8. **f**, **g** Schematic illustration of the polarization–relaxation process. F represents the dipole moment

To the best of our knowledge, impedance matching and EM dissipation performance are the two key factors that determine the EM absorption capacity of a material. The former determines the ratio of incoming EM waves to the EM waves that get into the interior of the absorber, whereas the latter determines the ability of the absorber to convert EM into Joule heat. Both performances are determined by the dielectric parameters for non-magnetic materials, namely $\varepsilon^{\prime}$ and $\varepsilon^{\prime\prime}$. The $\varepsilon^{\prime}$ value represents the ability to store electrical energy, and the $\varepsilon^{\prime\prime}$ value represents the dielectric loss ability, which results from the conductive and polarization–relaxation loss. To ascertain the EM absorption mechanism of GCs, we compared the permittivity of all GCs with that of C-cellulose and C-lignin. As shown in Fig. 6a, the $\varepsilon^{\prime}$ and $\varepsilon^{\prime\prime}$ values of C-lignin remained constant at about 3.8 and 0.1, respectively, over a wide frequency region (2–18.0 GHz). This indicates that C-lignin has a good impedance matching performance but a poor dielectric loss ability, which is caused by its poor conductive loss due to its intrinsically low crystallinity. This is the main reason underlying its poor EM absorption ability. The $\varepsilon^{\prime}$ and $\varepsilon^{\prime\prime}$ values of C-cellulose are observed in Fig. 6b to range from 29.4 to 12.5 and from 20.2 to 7.5, respectively. The increased crystallinity, evidenced by the enhanced diffraction peaks associated with (002) and (101) planes in the XRD spectra and the decreased $I_{D} /I_{G}$ value in the Raman spectrum, results in a higher $\varepsilon^{\prime}$ value, indicating an enhanced ability to store electrical energy. When the incident electromagnetic wave reaches the absorber, it generates a large amount of induced current within the absorber, resulting in more reflection and less transmission of EMs at the interface between air and the absorber/paraffin composites. As a result, an extremely high $\varepsilon^{\prime}$ value deteriorates the corresponding impedance matching performance via inducing excessive conductivity [64], implying that C-cellulose exhibits a poor EM absorption performance even if the material possesses a competitive ε″ value.

To elucidate on the permittivity variation for these GCs, the $\varepsilon^{\prime}$ and $\varepsilon^{\prime\prime}$ ratios of GC to C-cellulose are displayed in Fig. 6c, d (the corresponding measured dielectric values are shown in Fig. S8). We observe that all the $\varepsilon^{\prime}$ ratios of GCs were smaller than 1.0 except for GC-30, which possesses $\varepsilon^{\prime}$ ratios slightly larger than 1.0 at the local frequency region from 11.2 to 15.0 GHz. Such a phenomenon suggests a reduced $\varepsilon^{\prime}$ value compared to C-cellulose. The reduced $\varepsilon^{\prime}$ value implies enhanced impedance matching ability in GCs compared with C-cellulose, and the $\varepsilon^{\prime}$ ratios gradually increase from GC-4 to GC-30, suggesting that carbonized precursors with a higher cellulose content result in a larger $\varepsilon^{\prime}$ value. In addition, the $\varepsilon^{\prime\prime}$ ratios displayed in Fig. 6d follow the same trend as the $\varepsilon^{\prime}$ ratios, and the GC-30 has the largest $\varepsilon^{\prime\prime}$ ratios.

Next, we discuss the polarization loss of the GC materials. We note here that since the paraffin wax is non-magnetic and amorphous with ultralow dielectric parameters, its EM absorption performance is negligible. Regarding the polarization–relaxation process, the plot of $\varepsilon^{\prime}$ versus $\varepsilon^{\prime\prime}$ gives a single semicircle, normally denoted as the Cole–Cole semicircle, according to the classic Debye theory [65]. Specifically, the relative complex permittivity can be calculated as:

4 $$\varepsilon_{r} = \varepsilon_{\infty } + \frac{{\varepsilon_{s} - \varepsilon_{\infty } }}{1 + j2\pi f\tau } = \varepsilon^{\prime} - j\varepsilon$$

where $\varepsilon_{s}$, $\varepsilon_{\infty }$, $\tau$ are the static permittivity, the relative dielectric permittivity at a high-frequency limit, and polarization–relaxation time, respectively. After the separation of the real and imaginary parts, $\varepsilon^{\prime}$ and $\varepsilon^{\prime\prime}$ can be written as:

5 $$\varepsilon ^{\prime} = \varepsilon_{\infty } + \frac{{\varepsilon_{s} - \varepsilon_{\infty } }}{{1 + \left( {2\pi f} \right)^{2} \tau^{2} }}$$

6 $$\varepsilon ^{\prime\prime} = \varepsilon_{\infty } + \frac{{2\pi f\tau (\varepsilon_{s} - \varepsilon_{\infty } )}}{{1 + \left( {2\pi f} \right)^{2} \tau^{2} }}$$

Based on Eqs. (4) and (5), the $\varepsilon^{\prime} - \varepsilon^{\prime\prime}$ can be written as [66, 67]:

7 $$\left( {\varepsilon^{\prime} - \frac{{\varepsilon_{S} + \varepsilon_{\infty } }}{2}} \right)^{2} + \left( {\varepsilon s^{\prime \prime } } \right)^{2} = \left( {\frac{{\varepsilon_{S} - \varepsilon_{\infty } }}{2}} \right)^{2}$$

In this work, the effective absorption region was mainly located at a high-frequency range (i.e., 10–18.0 GHz) with thickness < 2.0 mm (Fig. 5e, f), so that the corresponding plots of $\varepsilon^{\prime}$ versus $\varepsilon^{\prime\prime}$ of the GCs were focused on this frequency range to deduce if the polarization effect plays an important role in EM dissipation. To elucidate on the polarization mechanism of GCs, the $\varepsilon^{\prime}$ versus $\varepsilon^{\prime\prime}$ curves of C-lignin and C-cellulose are also plotted as insets in Fig. 6a, b. First, two Cole–Cole semicircle profiles were observed in both C-lignin and C-cellulose, and the difference in these two semicircular profiles might be attributed to the difference in polarization relaxation intensity. We note here that the probable polarization at high frequency region (i.e., 10.0–18.0 GHz) results from dipole polarization [68].

Considering the fact that the formation of dipole-relaxation polarization in the presence of an applied microwave field depends on the presence of dipoles and their orientation, we propose a mechanism to explain the differences in the two Cole–Cole semicircle profiles. C-lignin and C-cellulose contain various defects, such as vacancies and dipolar bonds (e.g., C–O and C=OH). These defects have direction only if they are in the symmetrical graphitized area. In low-graphitized C-lignin, it has a high concentration of defects, which contribute to the polarization. Unlike C-lignin, C-cellulose has a higher graphitized area and it can induce dipole polarization when its defects concentrate in this area. The variation in the distributions and types of defects in the graphitized area greatly affects the polarization intensity and changes in the Cole–Cole semicircles (Fig. 6g). We note here that although the dipole-polarization behavior of GCs can be understood, the polarization intensity is difficult to quantify using current analytical techniques. As shown in Figs. 6 and S9, the dipole relaxation contributes to the EM dissipation and all GCs have two Cole–Cole semicircles.

We comment here that dipole relaxation behavior alone is insufficient to understand why the GC-8 sample had the broadest effective absorption band. To provide further insights, we discuss the role of conductive loss. The free-electron theory indicates that the conductive loss intensity is proportional to microwave conductivity ($\sigma$) [34]. In the microwave region, the energy is insufficient to excite more carriers and hasten their mobility, thus the microwave conductivity is equal to its static conductivity. According to the equivalent circuit theory [69], the static conductivity of carbon materials is proportional to its graphitization degree and nanostructure. Therefore, the conductive loss involves two steps: (1) materials self-respond to the electrical field and convert it into microcurrents, and (2) materials induce electronic transportation to form conductive loss. In step (1), the incident direction is important to determine the incident ratio, which strongly correlates to the carbon material nanostructure.

In GC materials, the characteristic nanosheet possesses structural characteristics of both central symmetry and axial symmetry, so that GC materials can maximize the use of electric field energy to drive the transport of electrons and form microcurrents. In GC-4, the poor graphitization results in a large resistivity, which leads to small conductive loss. In GC-5 and GC-8 that have both nanosheet and nanofiber structures, the nanosheet plays a key role in the formation of microcurrent, which tends to be transported through the highly conductive nanofibers. Since the nanofibers possess a larger aspect ratio, it prolongs the loss path and generates a considerable conductive loss. Therefore, GC-8 has a higher conductive loss than GC-5 owing to the high number density of nanofibers. GC-30, which only exhibits nanosheet structure, has high electrical utilization and small resistivity. Thus, the intensity of the EM-generated current in GC-30 is strong, which greatly shortens the propagation depth of an incident EM wave inside the absorber according to the skin depth theory and reduces the effective conductive loss. Based on the analysis of the EM parameters, the excellent EM absorption performance displayed by GC-8 can be attributed to the synergistic effect between the highly conductive carbon nanofibers and the graphite-like nanosheets with low conductivity, the interfacial polarization effect of these two structures, and the dipole polarization effect caused by the defects formed and multiple carbon heterocycles, are illustrated in Fig. S10 [70, 71].

### Hydrophobicity and Acid/Base Stability of GC-8

**Fig. 7:**
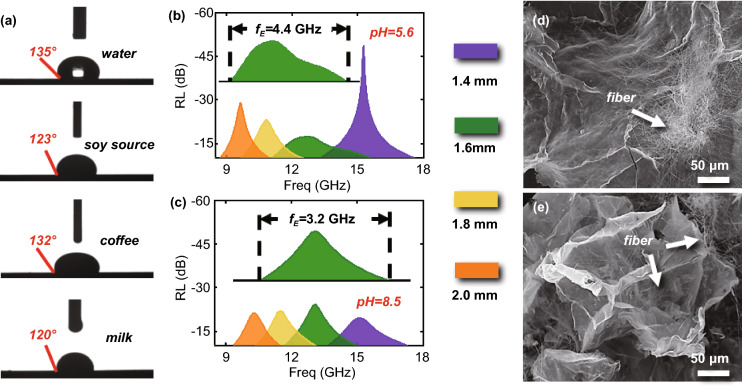
Environmental adaptability of GC-8. **a** Apparent contact angles of water, soy sauce, coffee, and milk on GC-8. **b**, **c** *f*_E_ curves of GC-8 after 7-day incubation in aqueous solutions with pH 5.6 and 8.5. The thickness of GC-8 film is < 2.0 mm. Inset shows the corresponding RL curve with the broadest *f*_E_. **d**, **e** SEM images for GC-8 samples after 7-day incubation in aqueous solutions with pH 5.6 and 8.5

In the final set of experiments, we sought to test the hydrophobicity and acid/base stability of GC materials under real environmental conditions. As shown in Fig. 7a, the apparent contact angle of water droplets on an EM film consisting of 15% GC-8 was measured to be ~ 135°, suggesting an enhanced hydrophobicity. We attribute the enhanced hydrophobicity to the significant removal of –O and –H during the carbonization process. In addition to water, the apparent contact angles of milk, coffee, and soy sauce on the film were greater than 120°, indicating a good omniphobicity. Furthermore, we tested the chemical stability of the GC materials by immersing GC-8 in either an acid solution with a pH value of 5.6 (which imitates the pH value of acid rain) or an alkaline solution with a pH of 8.5. The results in Figs. 7b, c and S11 indicate that after seven days of incubation in the acid or base solution, the EM dissipation performance of GC-8 remains unchanged in terms of broad $f_{{\text{E}}}$ and low ${\text{RL}}_{\min }$. Additional SEM imaging (Fig. 7d, e) does not show measurable changes in GC-8 after seven days of immersion, suggesting a good acid/alkaline stability. The above analysis demonstrates that the GC-8 samples exhibit outstanding EM absorption and good environmental adaptability, showing good potential for real-world application.

## Conclusions

This work reports the synthesis of graphitized carbon (GC)-based heterostructures consisting of nanofibers and nanosheets via pyrolysis of bamboo-derived lignocellulosic nanofibrils. Through manipulation of nanostructure shape and covalent bonds, an effective EM absorber with a broad $f_{{\text{E}}}$ (4.2 GHz) from 12.5 to 16.7 GHz at a thickness of 1.60 mm, as well as the thinnest required matching thickness (1.95 mm) for the lowest $\left| {{\text{RL}}_{{{\text{min}}}} } \right|$ of − 51.0 dB at 11.7 GHz, was obtained using precursors with a cellulose to lignin ratio of 8:1. Furthermore, the biomass-derived carbon materials exhibit enhanced hydrophobicity and acid/alkali resistance. Overall, the results reported in this work provide design principles for a new class of biomass-derived EM absorbers that possess exceptional EM dissipation abilities and exhibit good environmental adaptabilities for outdoor application.

## Supplementary Information

Below is the link to the electronic supplementary material.

Supplementary file1 (PDF 1355 KB)

## Data Availability

The datasets used or analyzed during the current study are available from the corresponding author on reasonable request.
