# Supplementary material for: Biomass-Derived Carbon Heterostructures Enable Environmentally Adaptive Wideband Electromagnetic Wave Absorbers
Source: Nanomicro Lett. 2021 Dec 4;14:11. doi: 10.1007/s40820-021-00750-z (PMC8643388; doi:10.1007/s40820-021-00750-z)
Supplement: Supplementary file 1 — Supplementary file1 (PDF 1355 KB) [file 40820_2021_750_MOESM1_ESM.pdf]

Supporting Information for

Biomass-Derived Carbon Heterostructures Enable Environmentally Adaptive Wideband Electromagnetic Wave Absorbers

Zhichao Lou[1], Qiuyi Wang[1], Ufuoma I. Kara[2], Rajdeep S. Mamtani[2], Xiaodi Zhou[1], Huiyang Bian[1], Zhihong Yang[3], Yanjun Li[1, *], Hualiang Lv[2, *], Solomon Adera[5, *], Xiaoguang Wang[2, 4, *]

[1]Jiangsu Co-Innovation Center of Efficient Processing and Utilization of Forest Resources, Nanjing Forestry University, Nanjing 210037, P. R. China

[2]Willian G. Lowrie Department of Chemical and Biomolecular Engineering, The Ohio State University, Columbus, OH, 43210, USA

[3]Institute of Materials Research and Engineering, Agency for Sciences, Technology and Research, Singapore

[4]Sustainability Institute, The Ohio State University, Columbus, OH, 43210, USA

[5]Department of Mechanical Engineering, University of Michigan, Ann Arbor, MI 48109, USA

*Corresponding authors. E-mail: nfcm2018@163.com (Yanjun Li); yexuexun5309@163.com (Hualiang Lv); sadera@umich.edu (Solomon Adera); wang.12206@osu.edu (Xiaoguang Wang)

Supplementary Tables and Figures

Table S1 Sample preparation parameters and nomenclature

Sample number	*p*-toluenesulfonic acid hydrolysis					pyrolysis
	Concentration	Temperature	Time	cellulose/lignin ratio	Nomenclature	Nomenclature
4	75 wt%	65 °C	60 min	4:1 w/w	LCNF-4	GC-4
5	40 wt%	80 °C	90 min	5:1 w/w	LCNF-5	GC-5
8	50 wt%	80 °C	60 min	8:1 w/w	LCNF-8	GC-8
30	80 wt%	80 °C	30 min	30:1 w/w	LCNF-30	GC-30

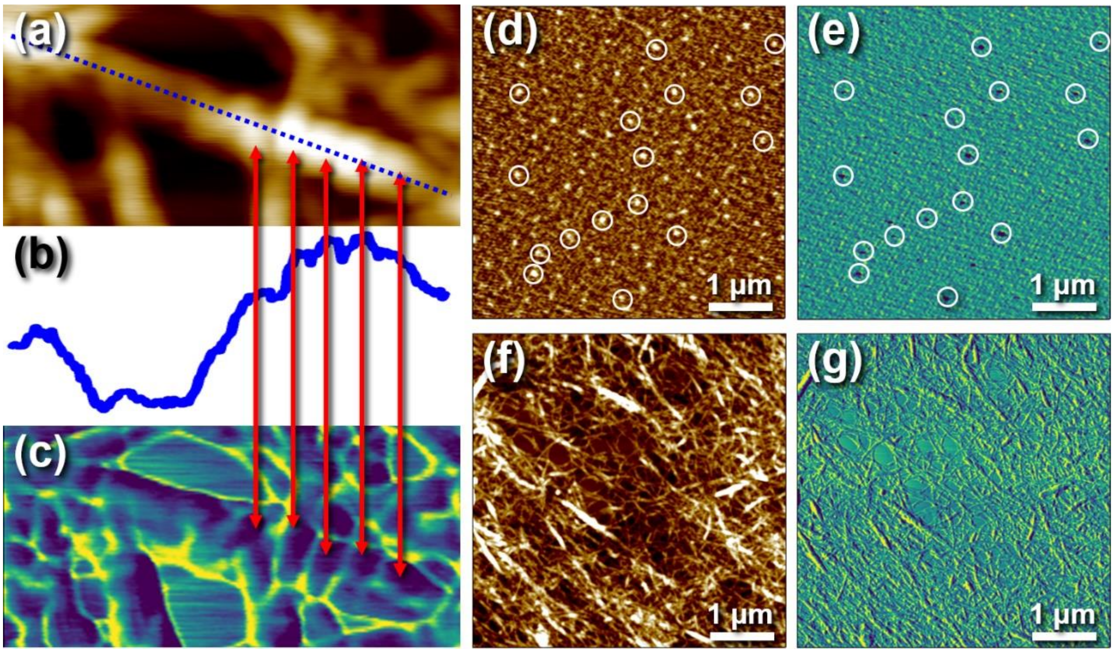

Fig. S1 a-c AFM topographic image, height curve, and AFM phasic image of the framed area in Figure 1(**i**) of the main text. **d-g** Representative AFM topographic and phasic images of pure lignin (**d, e**) and cellulose (**f, g**)

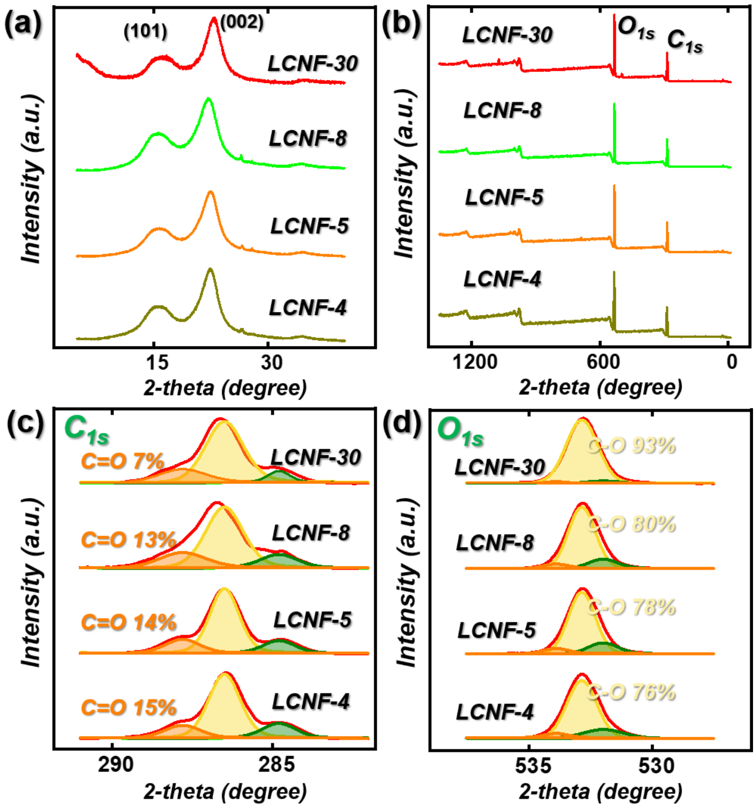

Fig. S2 (a) XRD curves of LCNF-4, LCNF-5, LCNF-8 and LCNF-30. **(b)** XPS survey curves of LCNF-4, LCNF-5, LCNF-8 and LCNF-30. **(c, d)** XPS curves of C_{1s} and O_{1s} with deconvoluted peaks

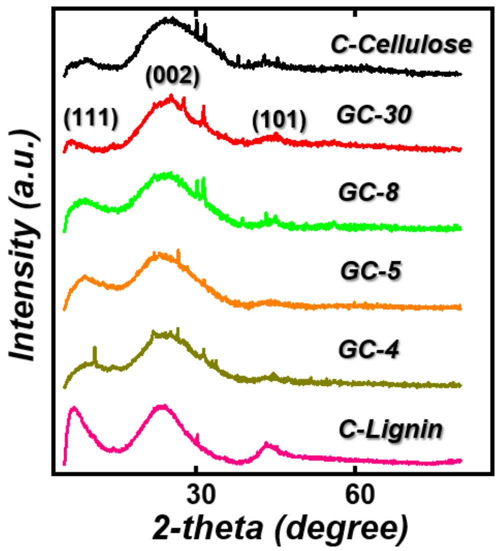

Fig. S3 XRD curves of the carbonized samples

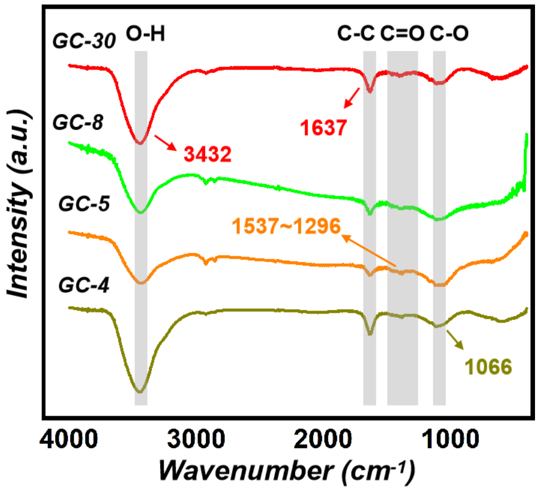

Fig. S4 FT-IR curves of the carbonized samples

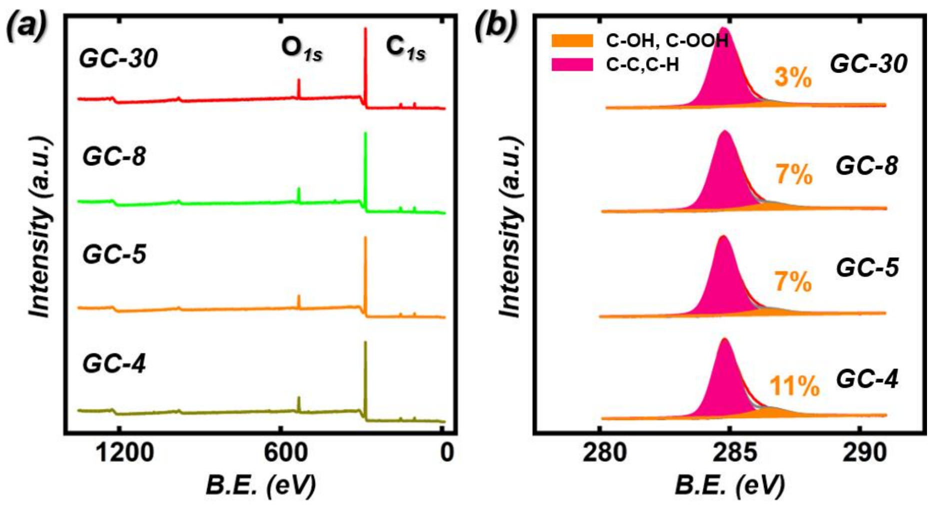

Fig. S5 XPS survey curves of the carbonized samples. **b** XPS curves of C_{1s} with deconvoluted peaks

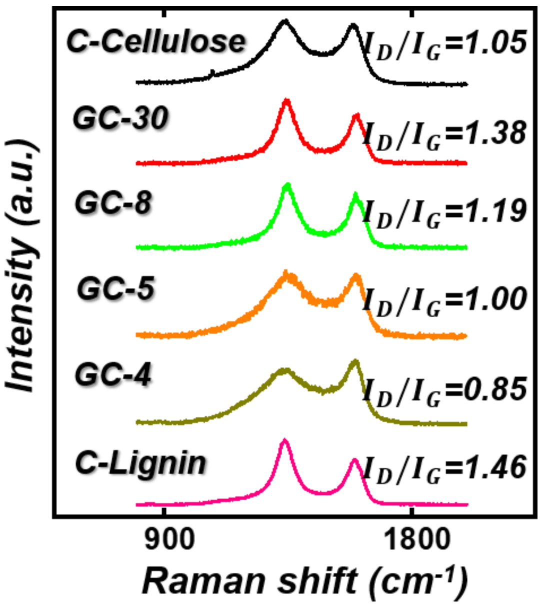

Fig. S6 Raman spectroscopy curves of the carbonized samples

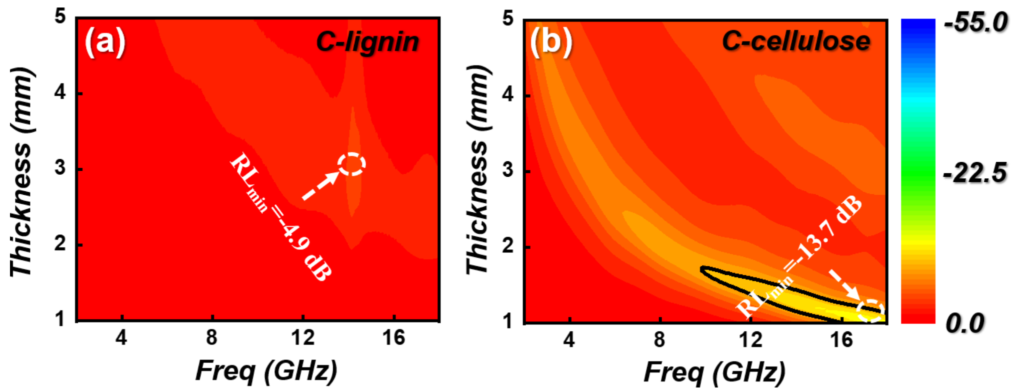

Fig. S7 2-D colored RL values of C-lignin and C-cellulose

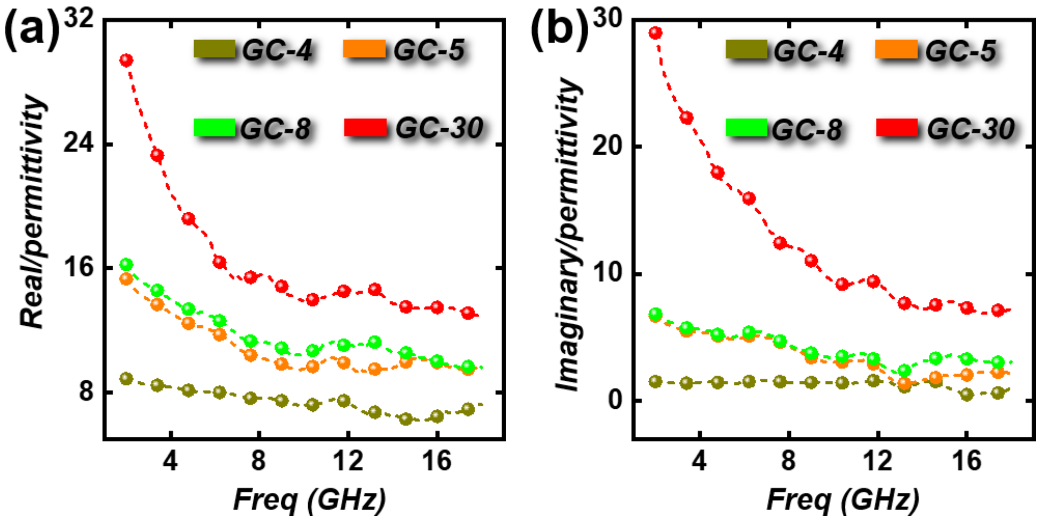

Fig. S8 Curves of **a** real part and **b** imaginary part of the permittivity of GCs

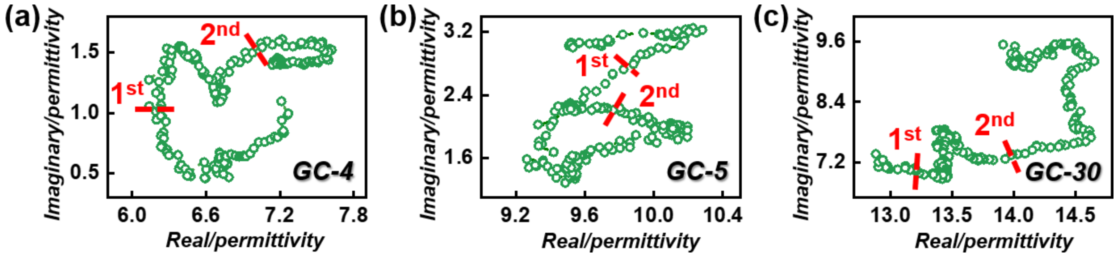

Fig. S9 Cole-Cole plots of GC-4, GC-5 and GC-30

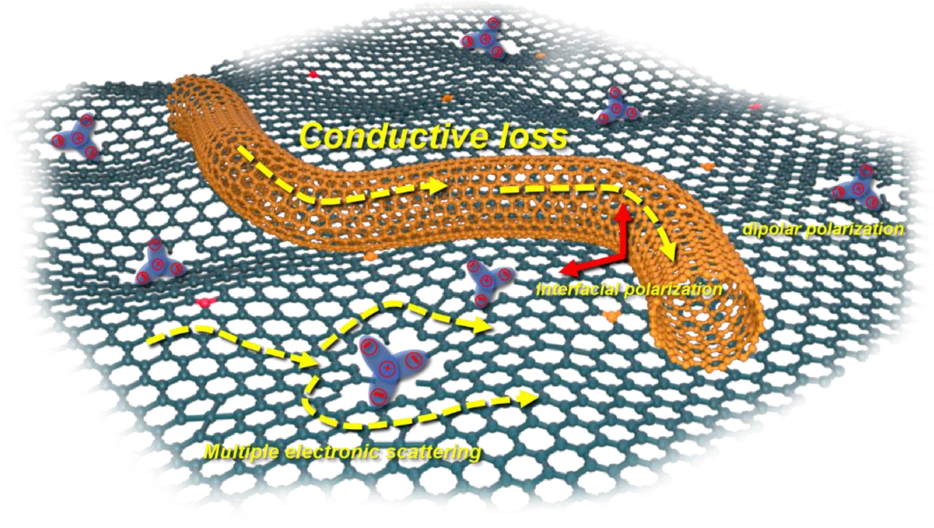

Fig. S10 Schematic illustration of electromagnetic loss mechanism

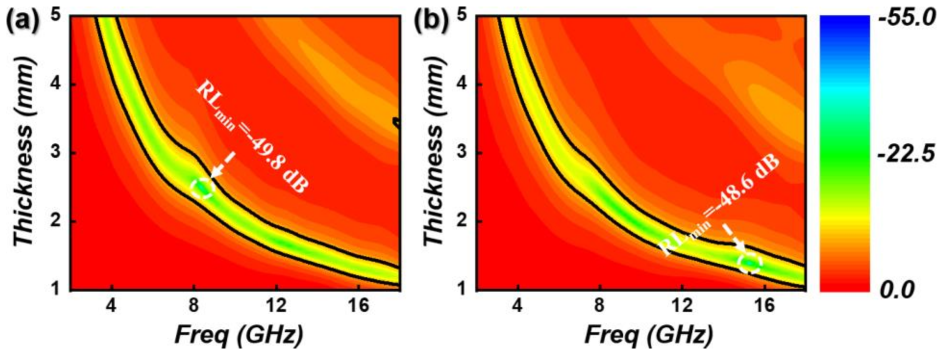

Fig. S11 2-D colored RL values of GC-8 after the 7-day incubation period in an aqueous solution of **a** pH=5.6 and **b** pH=8.5
